# Restoration of gallery forest patches improves recruitment of motacú palms (*Attalea princeps*) while diversifying and increasing wildlife populations

**DOI:** 10.1371/journal.pone.0250183

**Published:** 2021-04-29

**Authors:** Jo Peacock, Christopher M. Tonra, Johnathan King, G. Matt Davies

**Affiliations:** School of Environment and Natural Resources, The Ohio State University, Columbus, Ohio, United States of America; National University Comahue, ARGENTINA

## Abstract

Globally, forest-savanna mosaic landscapes are of significant conservation importance but have been widely impacted by human land-use. We studied how restoration, through cessation of long-term cattle grazing impacts (i) forest regeneration; (ii) forest understory structure and composition; and (iii) populations and diversity of large mammals and nocturnal birds, within naturally patchy gallery forests in the Beni Savannas of Bolivia. Comparing grazed and restored sites, we assessed the abundance and composition of tree functional types at different life stages (seedlings, saplings and adults), with focus on the region’s key palm species *Attalea princeps* (motacú). Additionally, we surveyed habitat structure and composition in the shrub and ground-layer, and monitored occurrence and encounter rates of large mammals and nocturnal birds along dusk and evening transects. We found evidence of lower recruitment of motacú palms on the grazed site and lower potential for natural motacú regeneration. Principal Components Analysis revealed forests on grazed sites had simpler, more open shrub-layers and altered ground-layer structure and composition including increased bare ground. Mammal species richness was greater on the restored site, and there were more declining, globally threatened and site-unique species. Species richness was similar for nocturnal birds within forests on both the grazed and restored site, but nearly all species tended to be encountered more frequently on the restored site. Our results suggest cattle negatively impact forest regeneration and alter the structure and composition of the shrub and ground layer with potential consequences for the diversity and abundance of wildlife. Our study represents one of only a handful completed in the Beni region of Bolivia to date. The Beni is currently under pressure from widespread, largely unregulated cattle ranching. Our results thus provide vital evidence to support development of restoration and conservation policy, and its integration with rangeland management in this threatened and critically understudied region.

## Introduction

Naturally occurring forest patches and gallery forests (hereafter: Natural Forest Patches; NFPs), like those found in savanna-mosaic landscapes, can have considerable importance for wildlife. NFPs provide habitat for various forest-dwelling species within open-matrix habitats [1–3] while supporting species requiring open and closed habitat simultaneously [4, 5], helping to maintain high landscape-level β-diversity [1–3, 6]. With more stable microclimates [7], NFPs also provide cover and shelter from extreme weather conditions and refuge from disturbance events like fires [8, 9]. Additionally, NFPs provision stepping-stone habitat [10], enabling the landscape-scale movement and dispersal of wildlife [6, 10, 11].

Unfortunately, savanna-mosaic landscapes have been widely and disproportionately affected by anthropogenic habitat degradation due to their importance for agriculture [12]. Today, these are considered some of the most rapidly disappearing and threatened habitats on earth [13, 14]. Cattle ranching is one of the primary agricultural activities that has impacted savanna-mosaics globally [12] and it continues to heavily influence the ecology of many regions. Particularly, Neotropical savanna-mosaics like the Brazilian Cerrado [15], Pantanal [16], Argentine Pampas [17] and Beni Savannas [18], where ranching on semi-natural rangeland remains central to local economies, but effects on wildlife and habitats are not well documented [19, 20]. Given the ecological importance of NFPs, it’s vital we better understand the impacts of ranching on their ecology and wildlife, including documenting how they recover following its alleviation. This will help inform much-needed conservation policy and strategies for ecosystem restoration and rewilding within these understudied regions [19].

The Beni Savannas are a 160 000km^2^ ecoregion in Northern Bolivia characterized by expansive savanna-grasslands, interspersed with small palm-forest islands and larger gallery forests that form more extensive, patchy networks along river corridors [21–23]. The Beni’s habitats are shaped by a multitude of abiotic conditions and disturbance factors including wildfire and seasonal flooding [21, 23, 24]. The region is home to a diverse mammal fauna, including many species known to utilize NFPs [25, 26]. These include top predators like puma *(Puma concolor)* and ocelot (*leopardus pardalis)*; species of international conservation concern like giant anteater *(Myrmecophaga tridactyla*; Vulnerable; [27]); and more common species like capybara *(Hydrochoerus hydrochaeris)* and howler monkeys *(Alouatta caraya)* [25, 26]. Nocturnal forest avifauna include the declining tropical screech owl *(Glaucidium brasilianum*), common pauraque *(Nyctidromus albicollis)* and great potoo (*Nyctibius grandis)* [25].

The Beni has been heavily modified by humans for thousands of years [28, 29]. Pre-Columbian indigenous populations constructed extensive earthworks here, including raised fields and fish weirs, that supported the persistence of complex societies [21, 28, 29]. Today, however, cattle ranching has replaced traditional agriculture and holds significant economic importance to local communities as the region’s primary industry [18]. Currently, Bolivian environmental policy places comparatively few constraints on Beni’s ranchers with regard to environmental protection or sustainability. Correspondingly, there has been a lack of research investment geared towards developing practical guidance and evidence to support ranching communities in implementing alternate land management strategies (i.e. focusing on potential benefits to the local economy, ranch productivity, market value of livestock and biodiversity), but see [30]. Ranching and associated land management, including the overuse and insufficient control of managed fire, are now believed to be degrading Beni’s habitats and impacting wildlife [19, 30–33]. However, the environmental effects of current stocking rates and ranching practices remain largely unknown and untested.

Recent research has linked ranching to habitat degradation in Beni, through negative impacts on the regeneration of, motacú palms (*Attalea princeps)* [32]. Motacú palms are a dominant component of Beni’s galley forests and forest islands, and play a key role in shaping their structure, microclimate and ecology [21, 31, 34, 35]. Their nuts form a vital component of the diet of many animals, including the critically endangered blue-throated macaw, *Ara glaucogularis* [31, 34].

In this study, we aimed to investigate how restoration, through cessation of long-term cattle grazing, influences the forest understory and wildlife in Beni’s gallery forests. Our specific objectives were to quantify restoration effects on i) forest regeneration, ii) forest understory structure and functional composition; and ii) populations and diversity of large mammals and nocturnal birds. Given the motacú’s ecological importance, we place particular emphasis on regeneration of this species.

## Methods

### Study site

**Fig 1:**
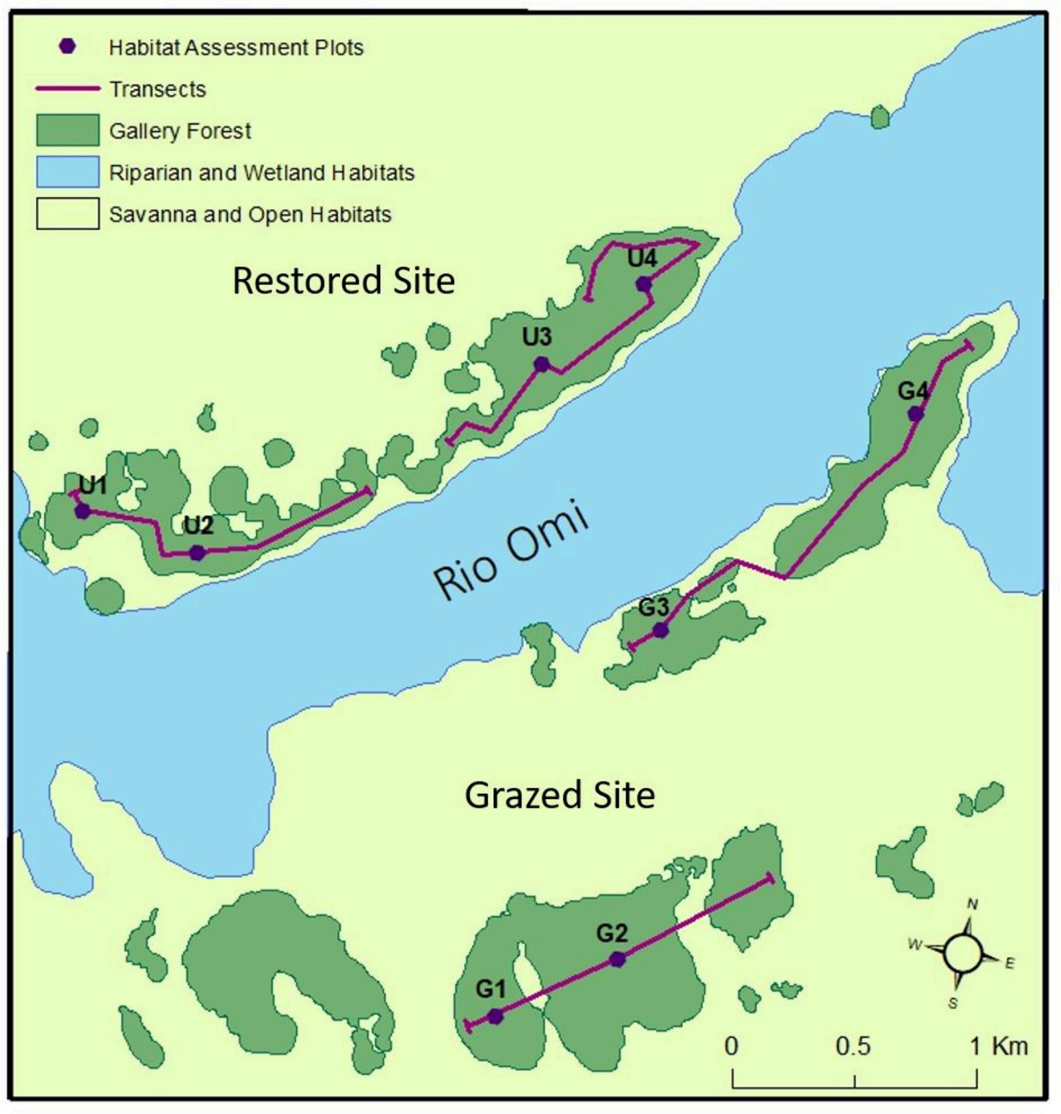
Map of the study area within Barba Azul Nature Reserve, showing the main sections of gallery forest within the restored and grazed sites (north and south of the Rio Omi respectively), and the locations of habitat assessment plots and wildlife line transects.

Our study took place in the galley forest islands of Barba Azul Nature Reserve, (hereafter: BANR; Fig 1), located within Bolivia’s Beni Savanna Ecoregion (13°45’S, 66°07’W). Beni’s gallery forests are diverse in composition, and vary in their pattern of distribution across the landscape [36, 37]. Those at the study site represent large patchy islands occurring on raised river levees, known locally as "alturas" [21]. They are typical in composition to the raised forests of both the flooded herbaceous savannahs, and palm-woodland sub-ecoregions which together cover approximately 40% of Beni’s land area [36, 37]. Mean annual temperatures in Beni range from 26–27°C, annual precipitation from 1300–2000 mm, with most falling in a distinct wet season running September-May [38]. Permits were not required for study, as data collection took place on a privately-owned reserve and collections were not required. Permissions to complete work on site were, however, obtained from the landowner, Asociación Civil Armonia.

Our study was implemented in two regions of BANR: Barba Azul North, a 3500 ha site on the northern banks of Rio Omi where cattle grazing has been excluded since 2009 (hereafter the restored site) and; Barba Azul South, a 1200 ha site on the southern banks of Rio Omi that was grazed at the time of survey by approximately 200–250 head of cattle (hereafter the grazed site). It is believed both sites have been grazed consistently for several decades, holding herds of between 250–350 head of cattle for at least the last 20–30 years. Historic grazing intensity prior to the last thirty years is assumed to have been relatively high with respect to the regional context [39]. This is due to the site’s close proximity to a meat-plane airfield, established on a neighbouring estancia soon after the onset of the cattle boom, which began in the 1950s. Although the restored site is now ungrazed, small groups of cows (20–30) occasionally abscond onto the site from nearby ranches for a few days at a time before being removed by reserve staff. It is therefore possible that this site is still subject to a small, infrequent amount of grazing pressure.

### Sampling methods

#### Habitat assessments

Fieldwork was completed during the dry season between July and August 2017. We carried out assessments at four locations (hereafter plots) within the gallery forests of each site (n = 8; Fig 1). Each consisted of a 100 m transect running parallel to either the north (grazed site) or south-facing (restored site) forest edge with a 20 X 20 m (400 m^2^) subplot centered on each transect midpoint. Within this subplot, we measured the diameter at breast height (dbh) of all adult trees (dbh > 10 cm at 1.3 m) and classified individuals as broadleaf’s, motacú’s, other palms or snags. The heights of adult motacú’s were measured using a Haglof Vertex IV Sonar Hypsometer. Saplings (dbh < 10 cm at 1.3 m and height > 30cm) were measured in the western half of the subplot (200 m^2^ area). We recorded sapling height for four groups; broadleaf’s, motacú, other palms and dead saplings, noting if saplings had obvious visible grazing damage (e.g. torn leaves, snapped/damaged stems, shorn stalks with ragged ends etc.). The latter was done qualitatively on a presence/absence basis, and we acknowledge this approach would not allow us to fully discount grazing impacts from other vertebrate or invertebrate herbivores. For broadleaf saplings, dbh was also recorded at 1.3 m. Where impossible due to height or structure in adults or saplings, dbh was taken under the first branch off the main stem. We did not record motacú sapling dbh as growth as juveniles is acaulescent (stemless) [40]. The line intercept method [41] was used to describe the shrub layer. We recorded cover of five plant functional groups (palms, broadleaf’s, woody shrubs, vines and ferns) along the entire transect to a maximum height of 2m above it. Five 4 m^2^ quadrats, regularly spaced along the transect were used to record the cover of bare ground, leaf litter, motacú nuts, deadwood, cow dung and three plant functional groups (forbs, ferns and graminoids). We also counted the absolute number of seedlings present for motacú (defined as per [40]) and woody plants (< 30 cm in height) within each quadrat. Seedlings of other palm species were not encountered.

#### Wildlife surveys—Mammals and nocturnal birds

We established two permeant line transects on each site (n = 4; Fig 1), each passing through at least two habitat assessment plots. Along each of these four transects, we carried out one dusk survey (for mammals) and one night survey (for mammals and nocturnal birds). Start and end times varied, but dusk surveys were generally completed between 17:30 and 18:45, night surveys, between 20:00 and 23:30. Dusk surveys lasted around one hour and night survey around two hours. For mammals, our total sampling effort was 8.11 km over 11 hrs. 53 mins. This equated to 4.25km walked over a total of 5 hours 51 mins on the grazed site and 3.86km walked over 6 hrs. 2 mins on the ungrazed site.

During dusk and night surveys, we walked slowly (~0.7 km per hour) and quietly, stopping often to scan and listen for activity. For all transects, we recorded start and end times, and total distance. After dark, we used a flashlight to scan for eye-shine and to identify animals. For mammals and nocturnal birds that were encountered visually (mammals and birds) or heard (birds), we recorded the time, distance from origin, species and number of individuals. To aid identification we referred to [42, 43] and sounds from [44].

### Data analysis

#### Forest regeneration

Plot level data was averaged across the four sampled plots for each site and the standard deviation (SD) calculated. This was done for seedling, sapling and adult tree counts as individuals per hectare (ha^-1^), and for calculations of sapling and adult tree basal area in m^2^ per ha^-1^. Basal area was calculated separately for each individual sapling (broadleaf only) and tree sampled using standard mathematical formulae (Area = π*radius^2^). Values were summed for each plot and converted to meters squared per hectare. To examine sapling height class distributions, sapling counts were plotted in 1m height-bands. To examine tree dbh class distributions, sapling and adult tree data were combined and plotted in 10cm bands. In order to facilitate this for motacú palms, acaulescent saplings were treated as the smallest “dbh” class. Finally, adult motacú’s were also split into 3m height bands. For all height bands and dbh classes, we report the with the mean count per ha^-1^ and SD at each site.

Following the approach developed by Volpato [45] we estimated the Natural Regeneration Index for motacú and broadleaf trees on the grazed and restored site. Natural Regeneration (NR) and Total Natural Regeneration (TNR) metrics (Eqs 1 and 2) can help examine the regeneration potential of species within forest ecosystems [45–50]. Similarly to other authors [46–50], we estimated NR in three height categories, (a) 1–2 m; (b) 2–3 m; and (c) >3 m. Considering only sapling height categories > 1 m ensures individuals included are in advanced stages of regeneration, having survived beyond critical periods of early mortality, and hence have increased probability of establishment [51]. In classification we considered only broadleaf saplings with dbh < 5cm, but included all motacú saplings counted.

$${NR}_{ij}=\frac{{RD}_{ij}+{RF}_{ij}}{2}$$ (1)

Where NR_ij_ = estimate of natural regeneration of the i-th species in the j-th height class in percent. RD_ij_ = relative density for the i^th^ species in the j^th^ height class of natural regeneration; RF_ij_ = relative frequency of the i^th^ species, in percentage, in the jth class of natural regeneration.

$${TNR}_{i}=\frac{\sum \left({NR}_{ij}\right)}{3}$$ (2)

Where TNR = total natural regeneration of the i-th species.

#### Forest understory structure and composition

Patterns in ground and shrub cover were assessed separately via two Principal Components Analyses (PCA; prcomp function, base R) [52]. We standardized percent cover values to a mean zero and unit variance to equalize weights in analyses and used the latent root criterion (eigenvalues > 1) to determine the number of interpretable axes [53]. Interpretation was achieved by examining factor loadings (FL) of each original variable included in the analysis on each axis. Only variables with FLs > 0.5 or < -0.5 were considered to have a pronounced effect on each axis. To allow graphical examination of the relationships between our study sites, and ground and shrub flora composition respectively, the centroid value and standard deviation for each site (grazed and restored) was plotted in multivariate space (ordiellispe function, base-R) [41].

#### Mammals and nocturnal birds

For each site, we divided the total observation period into twenty-four individual fifteen minute segments (up to a total of 6 hours) and constructed species accumulation curves using the “exact” method in function specaccum (Vegan package) [54]. For both mammals and nocturnal birds, we also calculated encounter rates per km^2^.

## Results

### Forest regeneration

**Fig 2:**
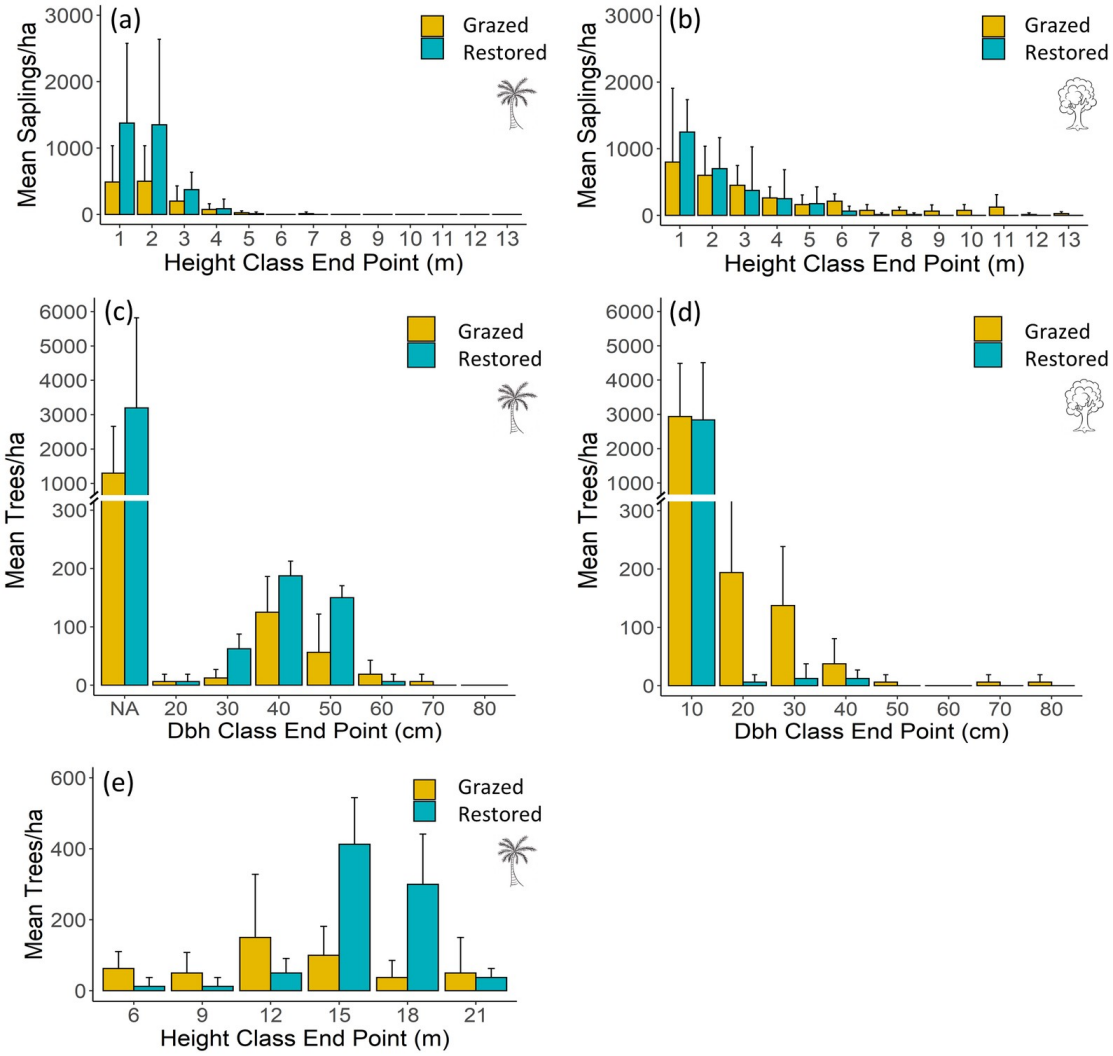
Average number of individuals per hectare in the grazed and restored sections of the reserve for (a) motacú saplings separated into 1m height classes; (b) broadleaf saplings separated into 1m height classes; (c) motacú saplings and adults separated into 10 cm dbh classes; (d) broadleaf saplings and adults separated into 10 cm dbh classes; and (e) motacú adult palms separated into 3m height classes. Note that in (c) acaulescent saplings are represented in the smallest dbh band and the band endpoint has thus been labelled NA on the plot axis. See methods for detail.

Total basal area of adult trees was similar between sites (Table 1a). Motacú made up the majority of total basal area on both sites, but motacú basal area was greater overall on the restored site. However, adult broadleaf tree density was greater than motacú on the grazed site (Table 1b). Despite differences in canopy composition, seedling density and composition was similar between sites (Table 1b), but over four times more saplings per ha^-1^ were recorded on the restored site (Table 1b). Broadleaf sapling counts were similar between sites, but motacú saplings were around two and a half times more abundant on the restored site (Table 1b). The basal area of broadleaf saplings was higher on the grazed site (Table 1a). The grazed site had more saplings in larger height classes (>5 m) and less in smaller height classes (1–2 m) compared to the restored site (Fig 2b). Likewise, there were fewer motacú in small height categories (1–4 m) on the grazed site compared to the restored site (Fig 2a). Dead and damaged saplings were only found on the grazed site, 1% of standing saplings were dead and 5% of live saplings had grazing damage.

**Table 1:** (a) Average basal area in m^2^ per hectare (Avg BA m^2^/ha); and (b) average number of individuals (bottom) per hectare (Avg Ind./ha) split by functional group for different tree life stages in grazed and restored areas of the reserve. Relative composition is reported where available.

Life Stage	Functional Group	Grazed			Restored		
**(a) Basal Area**:		Avg BA m^2^/ha	SD	% of total	Avg BA m^2^/ha	SD	% of total
Saplings	Broadleaf	3.3	2.3	-	0.8	0.8	-
	Motacú*	-	-	-	-	-	-
	Broadleaf Snag	0.06	0.1	-	0	0	-
	***Total***	***NA***			***NA***		
Adult Trees	Broadleaf	17.5	11.6	38%	1.88	1.3	4%
	Motacú	28.4	18.7	61%	46.8	5.7	94%
	Other Palm	0	0	0%	0.74	1.5	1%
	Broadleaf Snag	0.24	0.3	0.5%	0	0	0%
	Motacú Snag	0.35	0.7	0.8%	0.13	0.3	0.3%
	***Total***	***46*.*49***			***49*.*55***		
**(b) Number of Individuals**:		Avg Ind./ha	SD	% of total	Avg Ind./ha	SD	% of total
Seedlings	Broadleaf	27 875	11 665	72%	22 125	15 418	67%
	Motacú	10 875	3250	28%	10 750	13 763	33%
	***Total***	***38 750***			***32 875***		
Saplings	Broadleaf	2938	1551	69%	2838	1672	47%
	Motacú	1300	1363	30%	3200	2618	53%
	Broadleaf Snag	50	100	1%	0	0	0%
	***Total***	***4288***			***6038***		
Adult Tree	Broadleaf	388	171	60%	31	31.5	7%
	Motacú	225	95.7	40%	412	32.3	91%
	Other Palm	0	0		6	12.5	1%
	Broadleaf Snag	25	28.9	<1%	0	0	0%
	Motacú Snag	6	12.5	<1%	6	12.5	1%
	***Total***	***644***			***455***		

* Motacú saplings are acaulescent so basal area and hence relative composition could not be assessed.

For motacú, 12 400 individuals per ha^-1^ were recorded on the grazed site (88% seedlings, 10% saplings, 2% adults), 14 362 individuals per ha^-1^ on the restored site (75% seedlings, 22% saplings and 3% adults; Table 1). On both sites, motacú dbh size class distributions (Fig 1c) showed many acaulescent saplings (treated as the smallest dbh band in the figure) and a bell-shaped adult population centered ~40 cm (grazed site $\bar{\text{x}}=36.4$ cm, SD = 7.67 cm; restored site $\bar{\text{x}}=37.4$ cm, SD = 0.9 cm for adults only) with few large and few small individuals. Adult motacú height-class distributions were also bell-shaped (Fig 1e) with average adult motacú height tending to be greater on the restored site ($\bar{\text{x}}=14.3$; SD = 1) compared the grazed site ($\bar{\text{x}}=11.9$; SD = 3.52). On the restored site, broadleaf dbh classes (Fig 1d) show an inverse J distribution with large numbers of saplings, moderate numbers of small adults and few large adults. On the grazed site, dbh class distributions did not show an inverse J pattern. Here, we see many saplings, very few moderate sized adults and no adults in size classes >40 cm.

TNR for motacú was higher on the restored site compared to the grazed site (Table 2). The proportion of NR for motacú in the two smallest height categories was also higher on the restored site, but values for the largest size class were similar at both sites. In contrast, broadleaf TNR was higher on the grazed site. The proportion of broadleaf NR was higher in the two smallest height categories on the grazed site compared to the restored site, but values for the largest size class were similar at both sites.

**Table 2:** Proportion of Natural Regeneration (NR) in our three height classes of motacú and broadleaf saplings (NR1: 1–2 m, NR2: 2–3 m; and NR3: >3 m) and Total Natural Regeneration (TNR) on the a) grazed site and b) restored site.

	NR1%	NR2%	NR3%	TNR
a) Grazed				
Motacú	15.00	12.83	10.57	38.41
Broadleaf	18.33	20.50	22.76	61.59
b) Restored				
Motacú	17.98	20.24	9.39	47.62
Broadleaf	15.35	13.09	23.94	52.38

### Forest understory structure and composition

**Fig 3:**
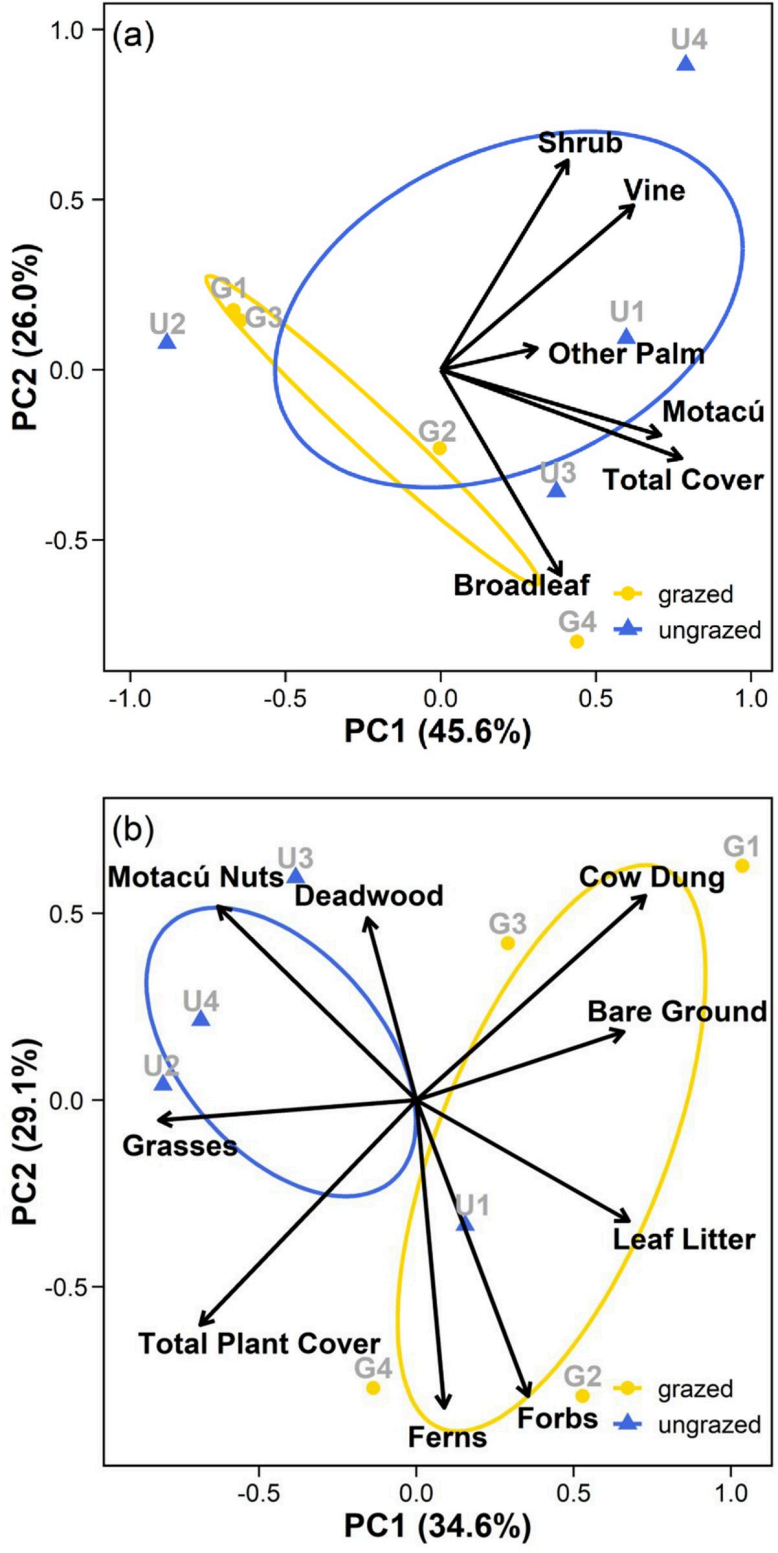
Principal Components Analysis (PCA), showing the first two principal components (PC1 and PC2) for a) shrub cover data and b) ground cover data in grazed and restored areas of the reserve. The variance explained by each axis is indicated on the axis label. Ellipses show standard deviation from the centroid value for each site (grazed and restored). Arrows represent the directional relationship of variables on axes. Arrow length is proportional to the relative strength of the relationship of each variable.

The first three axes of the ground and shrub layer PCA respectively explained 83% and 91% of the variation in each dataset. Factor loadings for individual variables on main axes are provided as supplementary information (S1 Table) For shrub cover PCA (Fig 3a), PC1 can be thought of as a gradient of shrub-layer density. Plots with higher axis scores on PC1 have denser understories dominated by motacú saplings. Those scoring low on PC1, sparser understories with few motacú saplings. PC2 can be thought of as a compositional gradient. Plots with higher axis scores on PC2 have understories dominated by shrubs and vines. Those scoring low on PC2, understories dominated by broadleaf saplings. Our analyses show spatial distinction of the sites. Restored plots tend towards higher scores on PC1 and exhibit greater variance in functional composition (indicated by the larger spread of the data and wider ellipse). Grazed plots tend towards the lower left quadrant of the PCA and exhibit narrower variance in composition (indicated by the smaller spread of data and narrower ellipse). For the ground cover PCA (Fig 3b), PC1 can be thought of as grazing impact gradient. Plots scoring high on PC1 having more cow dung, bare ground and leaf litter. Plots scoring low, more grasses, higher total plant cover and more fallen motacú nuts. PC2 relates to the cover of deadwood, forbs and ferns. Plots scoring high on PC2 can be thought of as having more deadwood. Plots scoring low on PC2, more forbs and ferns. The sites appear spatially segregated. Grazed plots sit higher on PC1 and exhibit more variance on PC2 compared to restored plots (larger ellipse and hence greater standard deviation from the mean) which tend to sit low on PC1 and high on PC2.

### Wildlife

**Fig 4:**
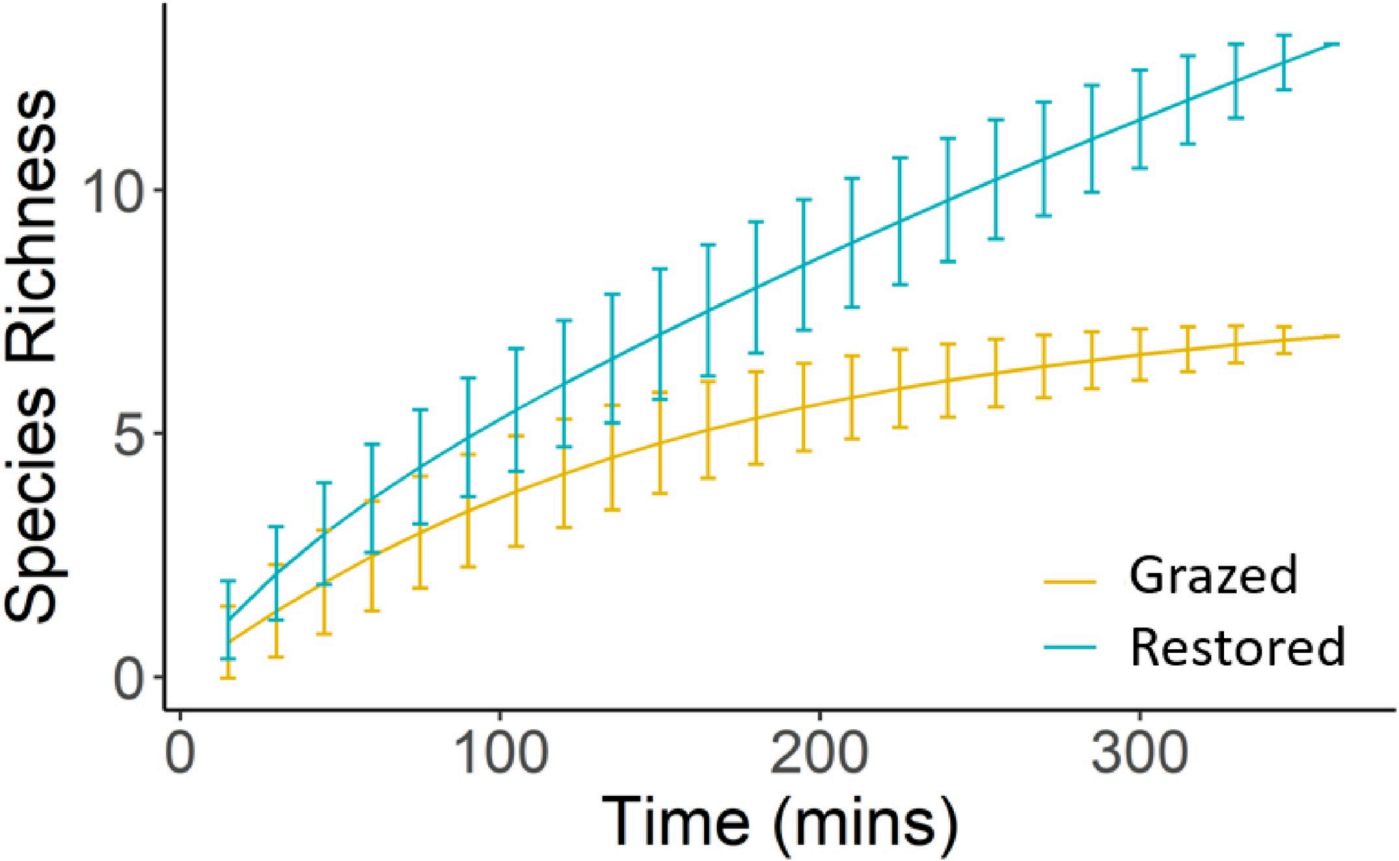
Mammalian species accumulation curves for dusk and night surveys carried out within the reserves’ grazed and restored gallery forest islands. Lines represent the mean species richness per cumulative 15m time band. Error bars represent the standard deviation from that mean.

We recorded 14 mammal species in total (Table 3). The restored site had higher mammalian species richness and more unique species. No IUCN red listed mammal species were recorded on the grazed site, but two declining species were. Of the shared mammal species, three were encountered more frequently on the restored site (brown agouti, black howler monkey and southern tamandua), and three more frequently on the grazed site (capybara, South-American coati and nine-banded armadillo). Our species accumulation analysis (Fig 4) shows steeper accumulation on the restored site with no evidence of approaching an asymptote. Accumulation on the grazed site was shallower and leveled out over the course of our study. We recorded seven nocturnal avian species in total (Table 3). Six species were recorded on the restored site, six on the grazed site. Each site had one unique species. Encounter rates for all species except tropical screech owl were generally 2–3 times higher on the restored site.

**Table 3:** Mammal and nocturnal bird species list and encounter rates per km (ER/km) on the grazed (GR) and restored (R) site.

Species	Scientific Name	IUCN Trend^3^	n GR	n R	ER/km GR	ER/km R	Diff. ER/km (R-GR)
**Mammals**:							
Brown Agouti	*Dasyprocta variegata*	DD	4	11	0.94	2.85	1.91
Capybara	*Hydrochoerus hydrochaeris*	↔	8	4	1.88	1.04	- 0.84
Collared Peccary	*Pecari tajacu*	↔	-	3	-	0.78	0.78
South American Coati	*Nasua nasua*	↓	17	1	4.00	0.26	- 3.74
Black Howler Monkey	*Alouatta caraya*	↓	15	18	3.53	4.66	1.13
Yellow Armadillo	*Euphractus sexcinctus*	↔	-	1	-	0.26	0.26
Nine-banded Armadillo	*Dasypus novemcinctus*	↔	4	2	0.94	0.52	-0.42
Gray Brocket Deer	*Mazama gouazoubira*	↓	-	1	-	0.26	0.26
Pampas Deer^1^	*Ozotoceros bezoarticus*	↓	-	1	-	0.26	0.26
Marsh Deer^2^	*Blastocerus dichotomus*	↓	-	1	-	0.26	0.26
Crab-eating Fox	*Cerdocyon thous*	↔	1	-	0.24	-	-0.24
Southern Tamandua	*Tamandua tetradactyla*	DD	1	1	0.24	0.26	0.20
Brazilian Porcupine	*Coendou prehensilis*	↔	-	1	-	0.26	0.26
Gray Four Eyed Opossum	*Philander opossum*	↔	-	1	-	0.26	0.26
**Nocturnal Birds**:							
Great Potoo	*Nyctibius grandis*	↓	1	3	0.48	1.36	0.88
Common Potoo	*Nyctibius griseus*	↓	2	-	0.95	-	-0.95
Ferruginous Pygmy Owl	*Glaucidium brasilianum*	↓	3	7	1.43	3.18	1.75
Tropical Screech Owl	*Megascops choliba*	↔	9	9	4.29	4.09	-0.20
Barn Owl	*Tyto alba*	↔	-	1	-	0.45	0.45
Common Pauraque	*Nyctidromus albicollis*	↓	7	17	3.33	7.73	4.40
Scissor-tailed Nightjar	*Hydropsalis torquata*	↔	1	4	0.48	1.81	1.33

^1^IUCN Status = Near Threatened.

^2^IUCN Status = Vulnerable.

^3^DD = Data Deficient, ↔ Stable, ↓ = Declining.

Includes current IUCN population trend [55], no of individuals recorded at each site (n) and the relative difference in encounter rates between sites (Diff. ER/km). A positive value indicates greater abundance on the restored site, a negative greater abundance on the restored site.

## Discussion

Our results suggest motacú and broadleaf regeneration did not differ greatly between the grazed and restored site at the seedling stage. However, the recruitment of motacú saplings and advanced motacú regeneration (i.e. TNR) appeared much lower on the grazed site. There were also few adult motacú’s in the smaller and larger adult height classes. Compared to the restored site, the grazed site had a simpler, more open and much less variable shrub-layer. The grazed site also had altered ground-layer structure and composition, including increased bare ground. Mammal species richness was greater on the restored site, and there were more declining, globally threatened and site-unique species. In contrast, species richness was similar for nocturnal birds between sites, most species tended to be encountered less frequently on the grazed site.

### Forest regeneration

We expected to see fewer broadleaf and motacú seedlings on the grazed site due to cattle impacts and, in the latter case, canopy differences between the sites, but this was not the case. Motacú and broadleaf seedling composition was relatively comparable between the sites and total seedling abundance was marginally higher on the grazed site. Cattle are often assumed to have negative impacts on tree seedling regeneration [e.g. 44]. However, in some tree taxa, cattle can facilitate regeneration by various direct and indirect processes. These include the removal of competitive plants, impacts on seed predators, exposure of microsites for germination, and enhanced seed dispersal [56–59]. In the case of motacú, it is feasible that cattle could play a role in facilitating seedling regeneration.

Cattle readily consume and disperse the fruits of *Attalea* palm species, depositing them in regurgitated heaps or dung piles [34, 60, 61]. The dung provides a mechanical barrier to bruchid beetle oviposition [62, 63] leading to reduced rates of infestation [34, 62] that may otherwise limit seedling growth in areas where fruits are densely deposited, e.g. carpets under parent trees [32, 64]. Cattle could thus facilitate motacú germination by reducing seed mortality and assisting with dispersal away from parent trees [65]. Motacú seeds are also dispersed by many native animals [34, 64, 66, 67]. Removal of the nutritious mesocarp by wildlife improves germination rates of *Attalea* species [67]. Our encounter rates for mammalian motacú dispersers, including coati, agouti and armadillo, are high compared to similar studies [e.g 68], even on the grazed site. BANRs forests are also known to be utilized by abundant foraging blue and yellow macaws, *Ara araruana* and other parrot species [69] that are key motacú dispersers [34]. As such, native seed dispersal may be relatively healthy on both of the sites we sampled.

Motacú sapling recruitment did not follow the same pattern as seedlings. Compared to the grazed site, motacú saplings were over twice as abundant on the restored site and constituted over twice the proportion of the sampled population. Advanced regeneration was also higher on the restored site (higher TNR), indicating motacú have greater regeneration potential on this site. Brazilian studies found similar [70] and slightly higher [40] proportions of saplings in motacú populations within forests where cattle were excluded over comparable periods [71, 72], suggesting cattle may negatively impact sapling recruitment. Over time, inhibited recruitment can lead to demographic bottlenecks in tree populations, threatening the long-term dynamics of forest composition, structure and function [73]. The lack of adult motacú in small size classes on our sites could reflect a population bottleneck caused by long-term grazing impacts on sapling recruitment. Comparable studies investigating cattle impacts on palm regeneration and population structure are rare [74]. Although Rivas (2005) [75], showed cattle had similar negative impacts on *Butia capitata* palm sapling regeneration, leading to an absence of young adult palms. However, tropical palms do often have u-shaped distributions [e.g. 74, 76 and 77], as saplings can persist for long periods in the understory and undergo rapid stem growth only when optimal light conditions prevail [74]. Consequentially, it may be difficult to detect anthropogenically-driven population bottlenecks from structural measures alone.

### Forest understory structure and composition

The shrub-layer was more open and less heterogeneous on the grazed site. The structure and composition of the forest floor also differed. Grazed plots had more bare ground, especially where recent evidence of cattle was present, and more leaf litter. Restored plots were dominated by grasses, fallen motacú nuts and deadwood. Our results suggest cattle may simplify (shrub-layer) and alter (ground cover) understory structure and composition within BANRs gallery forests. This is consistent with previous research in Beni’s palm forests, showing that cattle can damage and reduce understory cover [32], trample and compact forest soils to the detriment of plant growth [34] and affect palm health and fruiting productivity [32]. Our results also align with wider studies reporting structural and compositional simplification of the shrub layer associated with cattle trampling and browsing in other forest eco-types [78–81] and increases in bare soil in open and forested rangeland habitats [32, 82].

It is notable that there was one outlier in our shrub layer assessment (plot U2; Fig 3a). Although natural variability is to be expected within ecological systems, U2 appears to have surprisingly low shrub cover and abundance compared to other restored plots. Possible explanations include differences in soils within the forest [32], or legacy effects from past fire events [79]. Savanna fires typically extinguish on the forest edge as a consequence of differences in microclimate and fuels [7, 83], and generally only cause peripheral damage to Beni’s forest islands [22]. We avoided the forest edge in placement of our plots for that reason. However, plot U2 is located at a relatively narrow section of the gallery forest (Fig 1), immediately adjacent to the northern savanna vegetation. It is feasible the area may have been affected more than others by edge effects from historic grassland fire events.

The fate of the ground and shrub layers may be intertwined. Shrub-layer structure and composition can influence abiotic conditions in the understory (e.g. light penetration, microclimate), affecting plant growth and rates of decay on the forest floor [7, 84]. Microclimate, understory structure and the relative abundance of deadwood, leaf litter and detritus can be important factors determining the availability of insects and foraging habitat for insectivorous animals including nocturnal birds [85–89]. Likewise, palm fruit availability may influence the abundance of animals relaying on these food resources [90]. Grazing removal could thus facilitate shrub and ground-layer regeneration over relatively short time-scales with potential consequences for wildlife.

### Wildlife

For mammals, we recorded lower species richness and less unique, declining and globally threatened species on the grazed site. The restored site showed steeper species accumulation with no evidence of reaching an asymptote, while species accumulation was shallower on the grazed site and flattened over the course of our study. This suggests mammal diversity is higher on the restored site and that increased sampling effort would continue to yield new species records here, whereas on the grazed site it would not [91]. Not all species were encountered more frequently on the restored site. Capybara, coati and nine banded armadillo were encountered more frequently on the grazed site, and crab-eating fox were unique to it. With exception to coati, however, global populations of these species are stable [55], and thus may be less sensitive to pressures across their range.

The two globally threatened mammals observed on the restored site, marsh and pampas deer, are not forest species, but marshland and grassland specialists respectively [92, 93] and thus, are likely using the forest more opportunistically (i.e. for transit or cover). However, their presence is notable since, in combination with habitat loss, disease and parasite transmission from livestock, competition with cattle and hunting are major factors contributing to declines [92, 93]. As wide-ranging species [92, 93], marsh and pampas deer using the reserve still likely come into contact with cattle and other stressors in adjacent ranches. However, it’s feasible the reserves protection could be alleviating some pressure on the local population. Other species including peccary, agouti and brocket deer are also locally hunted as bush meat, but this is unlikely causing differences in occurrence between sites, as both areas have been protected from hunting for similar periods (2011 and 2009 on the grazed and restored site respectively). Grazed forests also had a less abundant nocturnal avifauna. With the exception of tropical screech owl, which are widely distributed and common in both undisturbed and human-disturbed habitats across their range [94], all species shared between the sites were encountered 2–3 times more frequently on the restored site. These include three of the four declining species encountered, great potoo, ferruginous pygmy owl and common pauraque. The grazed site did have one unique declining species, common potoo, while barn owl were unique to the restored site. Interestingly both site-unique species are associated more with other habitat types. Common potoo prefer wooded cerrado’s but utilize galley forest edges [95]. Barn owl may roost in forested areas, but specialize in foraging in open habitats [96]. As such, presence of these species might be influenced to a greater degree by composition of the surrounding matrix and proximity of our transects to such habitats.

Habitat structure, heterogeneity and composition can influence the variety, abundance and spatial distribution of wildlife, with species diversity and abundance tending to increase with structural and compositional complexity [97–100]. This aligns with our observation of greater mammalian richness and higher encounter rates for nocturnal birds on the restored site where the shrub layer was denser, more intact and relatively diverse in functional composition. However, the impact of specific forest structures may be species dependent. Some neotropical raptors, owls and nightjars are known to be more abundant in disturbed forests with open understories, possibly due to the increased prey detectability this affords, while others are associated with denser shrub-layers [78, 88]. Habitat degradation caused by cattle grazing could therefore have variable impact on the abundance and occurrence of different species on our sites. For example, tropical screech owls forage from low perches capturing prey on the ground or wing, which may make them more adaptable to open understory structures resulting from forest degradation [94]. Conversely, many ground-dwelling mammals like gray brocket deer tend to favor areas with dense understory cover [42] making them potentially less adaptable to cattle impacts in degraded forest understories.

An important limitation of our study is that, due to logistics, time and budget restrictions, wildlife sampling was restricted to a small number of line transects conducted during the dry season. Transect sampling may not be sufficient to capture all mammal or bird species present. Elusive creatures like big cats, smaller mammals like rodents and less vocal avian species can be missed by this approach [101–103]. Future survey efforts may consider including a variety of methods (e.g. Sherman traps, camera traps, track traps and avian mist netting) to expand the scope of results. Wildlife activity and cattle impacts may also vary seasonally due to the significant flooding that takes place across the region for several months of the year. For example, during the wet season, terrestrial wildlife and cattle may make more use of the forest islands to rest and shelter [30, 36]. Likewise, terrestrial animal movement may increase in the dry season with transient or dispersing species arriving from other forested regions across Beni [36]. Further sampling of grazing effects across these seasonal cycles is warranted.

Despite the apparent negative consequences for wildlife, it is also important to acknowledge that NFPs provide invaluable services to livestock producers in Beni [30]. For example, as shelter-beds for stock that can prevent death or exhaustion during extremes of hot or cold; as secluded areas for cows to calf; or, as places for herds to bed down in at night, or rest and dry their hooves during the wet season [20, 30, 32]. Identifying sustainable management regimes that allow maintenance of this economically-vital agricultural activity, while also protecting and restoring habitats for wildlife conservation, remains a critical research need.

## Conclusions

Our results suggest that cattle impart negative impacts on regeneration within BANRs gallery forests. Specifically, livestock hinder recruitment of motacú palms. Livestock removal may improve motacú regeneration potential by increasing the number of palm saplings in advance stages of development. Cattle also appear to simplify and alter the structure and composition of the shrub-layer and forest floor. This may have adverse consequences for wildlife, including effects on the abundance and diversity of mammals and nocturnal birds. On the restored site, motacú regeneration is more advanced and the shrub-layer is denser and more diverse. At the same time, nocturnal birds appear more abundant and mammal communities more diverse at least during the dry season when we were able to sample. Presumably, this is due to recent livestock removal. As such, the restored site may be moving towards a more desirable state in which natural motacú canopy regeneration can be supported by adequate recruitment in the understory, and where understory habitat structure and composition supports diverse, abundant faunal assemblages. Restoration, through grazing removal may thus facilitate regeneration and rewilding of the Beni’s gallery forests over relatively short time-scales.

## Supporting information

S1 Table Factor loadings for ground cover and shrub layer PCA variables. (DOCX)

S1 Dataset Dataset used for analyses (inclusive of supporting metadata). (XLSX)

## References

[1] Pardini R, Marques de Souza S, Braga-neto R, Metzger JP. The role of forest structure, fragment size and corridors in maintaining small mammal abundance and diversity in an Atlantic forest landscape. Biol Conserv. 2005;124: 253–266. 10.1016/j.biocon.2005.01.033

[2] Cáceres NC, Nápoli RP, Casella J, Hannibal W. Mammals in a fragmented savannah landscape in South-western Brazil. J Nat Hist. 2010;44: 491–512. 10.1080/00222930903477768

[3] Braga L, Diniz IR. Importance of habitat heterogeneity in richness and diversity of moths (Lepidoptera) in Brazilian Savanna. Environ Entomol. 2015;44: 499–508. 10.1093/ee/nvv026 26313955

[4] Brotons L, Herrando S, Martin J-L. Bird assemblages in forest fragments within Mediterranean mosaics created by wild fires. Landsc Ecol. 2005;19: 663–675. 10.1007/s10980-005-0165-2

[5] Di Blanco YE, Pérez IJ, Di Bitetti MS. Habitat Selection in Reintroduced Giant Anteaters: The Critical Role of Conservation Areas. J Mammal. 2015;96: 1024–1035. 10.1093/jmammal/gyv107

[6] Johnson MA, Saraiva PM, Coelho D. The role of gallery forests in the distribution of cerrado mammals. Rev Bras Biol. 1999;59: 421–427. 10.1590/s0034-71081999000300006

[7] Hoffmann WA, Jaconis SY, Mckinley KL, Geiger EL, Gotsch SG, Franco AC. Fuels or microclimate? Understanding the drivers of fire feedbacks at savanna-forest boundaries. Austral Ecol. 2012;37: 634–643. 10.1111/j.1442-9993.2011.02324.x

[8] Robinson NM, Leonard SWJ, Ritchie EG, Bassett M, Chia EK, Buckingham S, et al. Review: Refuges for fauna in fire-prone landscapes: Their ecological function and importance. J Appl Ecol. 2013;50: 1321–1329. 10.1111/1365-2664.12153

[9] Medri ÍM, Mourão G. A brief note on the sleeping habits of the giant anteater—Myrmecophaga tridactyla Linnaeus (Xenarthra, Myrmecophagidae). Rev Bras Zool. 2005;22: 1213–1215. 10.1590/s0101-81752005000400061

[10] Saura S, Bodin Ö, Fortin MJ. Stepping stones are crucial for species’ long-distance dispersal and range expansion through habitat networks. J Appl Ecol. 2014;51: 171–182. 10.1111/1365-2664.12179

[11] Cavalcanti SC, Marchini S, Zimmermann A, Gese EM, Macdonald DW. Jaguars, Livestock, and People in Brazil: Realities and Perceptions Jaguars, Livestock, and People in Brazil: Realities and Perceptions Behind The Conflict Behind The Conflict. Macdonald DW, Loveridge A, editors. The biology and conservation of wild felids. Oxford, United Kingdom: Oxford University Press; 2010. https://digitalcommons.unl.edu/icwdm_usdanwrc

[12] Sala OE, Chapin FS, Armesto JJ, Berlow E, Bloomfield J, Dirzo R, et al. Global biodiversity scenarios for the year 2100. Science (80-). 2000;287: 1770–1774. 10.1126/science.287.5459.1770 10710299

[13] Hoekstra JM, Boucher TM, Ricketts TH, Roberts C. Confronting a biome crisis: Global disparities of habitat loss and protection. Ecol Lett. 2005;8: 23–29. 10.1111/j.1461-0248.2004.00686.x

[14] Watson JEM, Jones KR, Fuller RA, Di Marco M, Segan DB, Butchart SHM, et al. Persistent Disparities between Recent Rates of Habitat Conversion and Protection and Implications for Future Global Conservation Targets. Conserv Lett. 2016;9: 413–421. 10.1111/conl.12295

[15] Klink CA, Machado RB. Conservation of the Brazilian Cerrado. Conserv Biol. 2005;19: 707–713. 10.1111/j.1523-1739.2005.00702.x

[16] Silva CJDA, Harris B, Tomas W, A GM. Safeguarding the Pantanal Wetlands: Threats and Conservation innitiatives. Conserv Biol. 2005;19: 714–720. 10.1111/j.1523-1739.2005.00708.x

[17] Minarro F, Bilenca D. The Conservation Status of Temperate Grasslands in Central Argentina. Fundacion Vida Silvestre Argentina. Buenos Aires, Argentina; 2008. http://awsassets.wwfar.panda.org/downloads/conservation_status_temperate_grasslands.pdf

[18] Aguilera R. La Ganaderia Beniana en Cifras. Federacion de Ganaderos del Beni y Pando. Trinidad, Bolivia; 2004. http://www.fegabeni.com.bo/images/documentos/LA GANADERIA BENIANA EN CIFRAS.pdf

[19] De Carvalho WD, Mustin K. The highly threatened and little known Amazonian savannahs. Nat Ecol Evol. 2017;1: 1–3. 28812663 10.1038/s41559-017-0100

[20] Hoogesteijn A. Cattle Ranching and Biodiversity Conservation as Allies in South America’s Flooded Savanas. Gt Plains Res. 2010;20: 37–50. Available: http://www.jstor.com/stable/23782174

[21] Langstroth R. Biogeography of the Llanos de Moxos: natural and anthropogenic determinants. Geogr Helv. 2012;66: 183–192. 10.5194/gh-66-183-2011

[22] Langstroth RP. Forest Islands in an Amazonian Savanna of Northeastern Bolivia. Ph.D. dissertation. Madison: University of Wisconsin. 1996. https://www.researchgate.net/publication/246214981_Forest_Islands_in_an_Amazonian_Savanna_of_Northeastern_Bolivia

[23] Mayle FE, Langstroth RP, Fisher RA, Meir P. Long-term forest-savannah dynamics in the Bolivian Amazon: Implications for conservation. Philos Trans R Soc B Biol Sci. 2007;362: 291–307. 10.1098/rstb.2006.1987 17255037 PMC2311431

[24] Hanagarth W. Acerca de la geoecología de las sabanas del Beni en el noreste de Bolivia. La Paz, Bolivia: Instituto de Ecología; 1993.

[25] Kingsbury J, McKenna A, Godsman K, McNeil D. Bolivia Expedition Report 2010. Glasgow, Scotland; 2010. https://drive.google.com/file/d/19pLBUswaqS6yZSOlweopS5Rl7XxVbMXO/view

[26] Kingsbury J, Ward R, MacDonald E, Thomson K, McCondichie A. Bolivia Expedition Report 2012. 2012; 1–112. Available: https://drive.google.com/file/d/19pLBUswaqS6yZSOlweopS5Rl7XxVbMXO/view

[27] Bertassoni M, Abba AM. Myrmecophaga tridactyla (Giant Anteater). e.T14224A47441961; 2014. http://maps.iucnredlist.org/map.html?id=14224. Downloaded on 29 July 2020.

[28] Denevan WM. The Aboriginal Cultural Geography of the Llanos. Berkeley: University of California Press; 1966. 10.1215/00182168-48.1.96

[29] Lombardo U, Iriarte J, Hilbert L, Ruiz-pérez J, Capriles JM, Veit H. Early Holocene crop cultivation and landscape modification in Amazonia. Nature. 2020;581: 190–195. 10.1038/s41586-020-2162-7 32404996 PMC7250647

[30] Mercado Callau LN, Boorsma T. Guia Practica Parra Ganaderia de Armonizacion; La Ganaderia Sostenible para el Beni. Santa Cruz de la Sierra, Bolivia; 2019. http://armoniabolivia.org/wp-content/uploads/2020/01/Guía-Ganadería-Sostenible-baja.pdf

[31] Yamashita C, de Barros YM. The Blue-throated Macaw Ara Glaucogularis: Characterization of its distinctive habitats in savannas of Beni, Bolivia. Rev Bras Ornitol—Brazilian J Ornithol. 1997;5: 10. Available: http://www4.museu-goeldi.br/revistabrornito/revista/index.php/BJO/article/view/0705

[32] Hordijk I, Meijer F, Nissen E, Boorsma T, Poorter L. Cattle affect regeneration of the palm species Attalea princeps in a Bolivian forest–savanna mosaic. Biotropica. 2019;51: 28–38. 10.1111/btp.12613

[33] Hesse AJ. The Blue-throated Macaw in the Wild: A Cause for Concern. Watchbird. 1997; 10–15. Available: https://journals.tdl.org/watchbird/index.php/watchbird/article/view/1107

[34] Baños-Villalba A, Blanco G, Díaz-Luque JA, Dénes F V., Hiraldo F, Tella JL. Seed dispersal by macaws shapes the landscape of an Amazonian ecosystem. Sci Rep. 2017;7: 1–12. 28785083 10.1038/s41598-017-07697-5 PMC5547140

[35] Moraes MR, Zenteno-Ruiz FS. El género Attalea (Arecaceae) de Bolivia: Afinidades con sistemas ecológicos regionales. Rev Peru Biol. 2017;24: 273–282. 10.15381/rpb.v24i3.13913

[36] Larrea-Alcázar DM, Embert D, Aguirre LF, Ríos-Uzeda B, Quintanilla M, Vargas A. Spatial patterns of biological diversity in a neotropical lowland savanna of northeastern Bolivia. Biodivers Conserv. 2011;20: 1167–1182. 10.1007/s10531-011-0021-4

[37] Larrea-Alcázar DM, López RP, Quintanilla M, Vargas A. Gap analysis of two savanna-type ecoregions: A two-scale floristic approach applied to the Llanos de Moxos and Beni Cerrado, Bolivia. Biodivers Conserv. 2010;19: 1769–1783. 10.1007/s10531-010-9802-4

[38] Borghetti F, Barbosa E, Ribiero L, Ribiero JF, Machado Teles Walter B. South American Savannas. In: Scogings PF, Sankaran M, editors. Savanna Woody Plants and Large Herbivores. Chichester, West Sussex, UK: John Wiley and Sons Ltd; 2019. pp. 77–122.

[39] Denevan WM. Cattle Ranching in the Mojos Savannas of Northeastern Bolivia. Yearb Assoc Pacific Coast Geogr. 1963;25: 37–44. Available: https://www.jstor.org/stable/24042297

[40] Giroldo AB, Nascimento ART, Silva PPF, Pinho GV JR. Population Structure and Density of Attalea phalerata Mart. Ex Spreng (Arecaceae) in a Semideciduous Forest. Rev Arvore. 2012;36: 637–645. Available: https://www.scielo.br/pdf/rarv/v36n4/a06v36n4.pdf

[41] Canfield R. Application of the Line Interception Method in Sampling Range Vegetation. J For. 1941;39: 388–394. Available: https://academic.oup.com/jof/article-abstract/39/4/388/4706387

[42] Emmons LH, Freer F. Neotropical rainforest mammals: a field guide. 2nd ed. Chicago, IL: University of Chicago Press; 1997.

[43] Herzog SK, Terrill RS, Jahn AE, Remsen J V, Maillard O, Garcia-Soliz VH, et al. Birds of Bolivia, Field Guide. 1st ed. Santa Cruz, Bolivia: Asociation Armonia; 2016.

[44] Mayer S. Birds of Bolivia 2.22. CD ROM. Birdsongs International. Enschede, The Netherlands. 2010.

[45] Volpato MML. Regeneração natural em uma floresta secundária no domínio de mata atlântica: uma análise fitossociológica. Federal University of Viçosa, Viçosa. 1994.

[46] Lima ES, Felfili JM, Marimon BS, Scariot Ae. Diversidade, estrutura e distribuição espacial de palmeiras em um cerrado sensu stricto no Brasil Central—DF. Rev Bras Bot. 2003;26: 361–370. 10.1590/S0100-84042003000300009

[47] Da Silva WC, Marangon LC, Ferreira LRC, Feliciano ALP JR, Costa RF. Estudo da regeneração natural de espécies arbóreas em fragmento de Floresta Ombrófila Densa, Mata das Galinhas, no município de Catende, zona da mata sul de Pernambuco. Ciência Florestal, St Maria. 2007;17: 321–331. 10.5902/198050981964

[48] Ebert A, Teixeira LR, Da Silva AZC, Da Costa RB. Natural Regeneration in Tropical Secondary Forest in Southern Amazonia. Open J For. 2014;4: 151–160. 10.4236/ojf.2014.42021

[49] De Araújo AB JR, Costa RF, Lopes YS, Celestino PCG, De Almeida ACS, Chaves L de F de CC. Dynamics of Natural Regeneration in a Fragment of Dense Ombrophilous Forest in Urban Area. J Exp Agric Int. 2018;27: 1–15. 10.9734/JEAI/2018/44621

[50] Junior BHM, Haridasan M. Comparação da vegetação arbórea e características edáficas de um cerradão e um cerrado sensu stricto em áreas adjacentes sobre solo distrófico no leste de Mato Grosso, Brasil. Acta Bot Brasilica. 2005;19: 913–926. 10.1590/S0102-33062005000400026

[51] Lima RB de A, Marangon LC, Freire FJ, Feliciano ALP, Soares de Silva RK. Potencial regenerativo de espécies arbóreas em fragmento de Mata Atlântica, Pernambuco, Brasil. Rev Verde Agroecol e Desenvolv Sustentável. 2017;12: 666–673. 10.18378/rvads.v12i4.5002

[52] R Core Team. R: A Language and Environment for Statistical Computing. 3.6.2. R Foundation for Statistical Computing, Vienna, Austria. https://www.R-project.org/. Vienna, Austria: R Foundation for Statistical Computing; 2020.

[53] McCune B, Grace JB. Analysis of Ecological Communities. Gleneden Beach, Oregon: MjM Software Design; 2002.

[54] Oksanen J, Blanchet FG, Friendly M, Kindt R, Legendre P, Mcglinn D, et al. Package “Vegan”—Community Ecology Package: Ordination, Diversity and Dissimilarities. R package. Version 2.5–6. 2019. pp. 1–297. https://cran.r-project.org/web/packages/vegan/vegan.pdf

[55] IUCN. The IUCN Red List of Threatened Species. Version 2020–2. 2020. https://www.iucnredlist.org/

[56] Janzen DH. Differential Seed Survival and Passage Rates in Cows and Horses, Surrogate Pleistocene Dispersal Agents. Oikos. 1982;38: 150–156. 10.2307/3544014

[57] Fortuny X, Carcaillet C, Chauchard S. Selective and taxon-dependent effects of semi-feral cattle grazing on tree regeneration in an old-growth Mediterranean mountain forest. For Ecosyst. 2020;7. 10.1186/s40663-020-00222-7

[58] Goheen JR, Palmer TM, Keesing F, Riginos C, Young TP. Large herbivores facilitate savanna tree establishment via diverse and indirect pathways. J Anim Ecol. 2010;79: 372–382. 10.1111/j.1365-2656.2009.01644.x 20039982

[59] Dufour-Dror JM. Influence of cattle grazing on the density of oak seedlings and saplings in a Tabor oak forest in Israel. Acta Oecologica. 2007;31: 223–228. 10.1016/j.actao.2006.11.003

[60] Scariot A. Seed Dispersal and Predation of the Palm Acrocomia aculeata. Principes. 1998;42: 5–8.

[61] Yamashita C. Anodorhynchus macaws as followers of extinct megafauna: an hypothesis. Ararajuba. 1997;5: 176–182. Available: http://www.revbrasilornitol.com.br/BJO/article/view/0710

[62] Rios RS, Pacheco LF. The effect of dung and dispersal on postdispersal seed predation of Attalea phalerata (Arecaceae) by bruchid beetles. Biotropica. 2006;38: 778–781. 10.1111/j.1744-7429.2006.00209.x

[63] Quiroga-Castro VD, Roldan AI. The Fate of Attalea phalerata (Palmae) Seeds Dispersed to a Tapir Latrine. Biotropica. 2001;33: 472–477. 10.1111/j.1744-7429.2001.tb00200.x

[64] Pimentel Savio D, Tabarelli M. Seed Dispersal of the Palm Attalea oleifera in a Remnant of the Brazilian Atlantic Forest. Biotropica. 2004;36: 74–84. 10.1111/j.1744-7429.2004.tb00298.x

[65] Choo J, Juenger TE, Simpson BB. Consequences of frugivore-mediated seed dispersal for the spatial and genetic structures of a neotropical palm. Mol Ecol. 2012;21: 1019–1031. 10.1111/j.1365-294X.2011.05425.x 22229743

[66] Desbiez LJA, Borges PAL. Density, habitat selection and observations of South American Coati Nasua nasua in the central region of the Brazilian Pantanal Wetland. Small Carniv Conserv. 2010;42: 14–18. Available: https://www.researchgate.net/profile/Arnaud_Desbiez/publication/265484453_Density_habitat_selection_and_observations_of_South_American_Coati_Nasua_nasua_in_the_central_region_of_the_Brazilian_Pantanal_wetland/links/558beebf08ae591c19d915fd.pdf

[67] Anderson AB, May PH, Balick MJ. The subsidy from nature: Palm forests, peasantry, and development on an Amazon frontier. New York, USA: Columbia University Press; 1991.

[68] Desbiez ALJ, Bodmer RE, Santos SA. Wildlife habitat selection and sustainable resources management in a Neotropical wetland. Int J Biodivers Conserv. 2009;1: 11–20. Available: https://academicjournals.org/journal/IJBC/article-full-text-pdf/DAAF3BB2435.pdf

[69] MacDonald E. Foraging Ecology and Population Size of the Critically Endangered Blue-throated Macaw (Ara glaucogularis) and the Sympatric Blue and Yellow Macaw (Ara ararauna) on the Barba Azul Reserve, Bolivia. Unpublished Masters Thesis. University of Glasgow. 2012. http://armoniabolivia.org/wp-content/uploads/2016/07/01_2012-MacDonald_GlasgowUniversity.pdf

[70] Bonato Negrelle RR. Estrutura populacional e Potencial de Regeneacao De Attalea phalerata Mart. ex Spreng. (Acuri). Ciência Florestal, St Maria. 2013;23: 727–734. Available: https://www.scielo.br/scielo.php?pid=S1980-50982013000400727&script=sci_abstract

[71] Brandão LG, Antas P de TZ, Flamarion B. de Oliveira L, Pádua MTJ, Pereira N da C, Valutky WW. Plano de Manejo da Reserva Particular de Patrimônio Natural do SESC Pantanal. Rio de Janeiro, Brazil; 2011. https://www.researchgate.net/publication/267267555_Plano_de_Manejo_da_Reserva_Particular_de_Patrimonio_Natural_do_SESC_Pantanal_2_edicao_-revista_e_atualizada

[72] IEF. Plano de Manejo Parque Estadual do Pau Furado. Uberlandia, Minas Gerais; 2011. http://paufurado.blogspot.com/p/biblioteca.html

[73] Spies TA. Forest Structure: A Key to the Ecosystem: in Trofymow J.A. and MacKinnon A., editors. Proceedings of a workshop on Structure, Process, and Diversity in Successional Forests of Coastal British Columbia,. 1998;72(2): 34–39. Available: https://andrewsforest.oregonstate.edu/sites/default/files/lter/pubs/pdf/pub2564.pdf

[74] Montúfar R, Anthelme F, Pintaud J-C, Balslev H. Disturbance and Resilience in Tropical American Palm Populations and Communities. Bot Rev. 2011;77: 426–461. 10.1007/s12229-011-9085-9

[75] Rivas M. Desafíos y alternativas para la conservación in situ de los palmares de Butia capitata (MART.) Becc. Agrociencia. 2005;IX: 161–168.

[76] Felfili JM. Diameter and height distributions in a gallery forest tree community and some of its main species in central Brazil over a six-year period (1985–1991). Rev Bras Bot. 1997;20: 155–162. 10.1590/s0100-84041997000200006

[77] Dantes A, Lira-Guedes A, Guedes M, Piedade M, Batista A. Population Dynamics of Attalea excelsa (Arecaceae) in flooplain forest of the Amazon Estuary. J Trop For Sci. 2020;32: 105–113. 10.26525/jtfs32.2.105

[78] Piana RP, Marsden SJ. Impacts of cattle grazing on forest structure and raptor distribution within a neotropical protected area. Biodivers Conserv. 2014;23: 559–572. 10.1007/s10531-013-0616-z

[79] Tasker EM, Bradstock RA. Influence of cattle grazing practices on forest understorey structure in north-eastern New South Wales. Austral Ecol. 2006;31: 490–502. 10.1111/j.1442-9993.2006.01597.x

[80] Varga A, Demeter L, Ulicsni V, Öllerer K, Biró M, Babai D, et al. Prohibited, but still present: local and traditional knowledge about the practice and impact of forest grazing by domestic livestock in Hungary. J Ethnobiol Ethnomed. 2020;16: 51. 10.1186/s13002-020-00397-x 32912227 PMC7488016

[81] Belsky AJ, Blumenthal DM. Effects of Livestock Grazing on Stand Dynamics and Soils in Upland Forests of the Interior West. Conserv Biol. 1997;11: 315–327. 10.1046/j.1523-1739.1997.95405.x

[82] Fuhlendorf SD, Harrell WC, Engle DM, Hamilton RG, Davis CA, Leslie DM. Should heterogeneity be the basis for conservation? Grassland bird response to fire and grazing. Ecol Appl. 2006;16: 1706–1716. 10.1890/1051-0761(2006)016[1706:shbtbf]2.0.co;2 17069365

[83] Hoffmann WA, Geiger EL, Gotsch SG, Rossatto DR, Silva LCR, Lau OL, et al. Ecological thresholds at the savanna-forest boundary: How plant traits, resources and fire govern the distribution of tropical biomes. Ecol Lett. 2012;15: 759–768. 10.1111/j.1461-0248.2012.01789.x 22554474

[84] Kovács B, Tinya F, Ódor P. Stand structural drivers of microclimate in mature temperate mixed forests. Agric For Meteorol. 2017;234–235: 11–21. 10.1016/j.agrformet.2016.11.268

[85] Morante-Filho JC, Arroyo-Rodríguez V, Lohbeck M, Tscharntke T, Faria D. Tropical forest loss and its multitrophic effects on insect herbivory. Ecology. 2016;97: 3315–3325. 10.1002/ecy.1592 27911998

[86] Sandström J, Bernes C, Junninen K, Lõhmus A, Macdonald E, Müller J, et al. Impacts of dead wood manipulation on the biodiversity of temperate and boreal forests. A systematic review. J Appl Ecol. 2019;56: 1770–1781. 10.1111/1365-2664.13395

[87] Jucker T, Jackson TD, Zellweger F, Swinfield T, Gregory N, Williamson J, et al. A Research Agenda for Microclimate Ecology in Human-Modified Tropical Forests. Front For Glob Chang. 2020;2: 1–11. 10.3389/ffgc.2019.00092

[88] Barros OG, Cintra R. The effects of forest structure on occurrence and abundance of three owl species (Aves: Strigidae) in the Central Amazon forest. Zoologia. 2009;26: 85–96. 10.1590/S1984-46702009000100014

[89] English PA, Green DJ, Nocera JJ. Stable Isotopes From Museum Specimens May Provide Evidence of Long-Term Change in the Trophic Ecology of a Migratory Aerial Insectivore. Front Ecol Evol. 2018;6: 1–13. 10.3389/fevo.2018.00014

[90] Haugaasen T, Peres CA. Vertebrate responses to fruit production in Amazonian flooded and unflooded forests. Biodivers Conserv. 2007;16: 4165–4190. 10.1007/s10531-007-9217-z

[91] Oksanen J. Vegan: ecological diversity. R Packag Version 24–4. 2017;1: 11. https://cran.r-project.org/package=vegan

[92] Duarte JM., Varela D, Piovezan U, Beccaceci M, Garcia J. Blastocerus dichotomus. In: The IUCN Red List of Threatened Species: e.T2828A22160916. [Internet]. 2016. 10.2305/IUCN.UK.2016-1.RLTS.T2828A22160916.en.

[93] González S, Jackson III J, Merino M. Ozotoceros bezoarticus. In: The IUCN Red List of Threatened Species: e.T15803A22160030. [Internet]. 2016. 10.2305/IUCN.UK.2016-1.RLTS.T15803A22160030.en.

[94] Ong G. Tropical Screech-Owl (Megascops choliba), version 1.0. In Birds of the World (Schulenberg T. S., Editor). Cornell Lab of Ornithology, Ithaca, NY, USA. https://doi-org.proxy.lib.ohio-state.edu/10.2173/bow.trsowl.01. 2020.

[95] Various. Common Potoo (Nyctibius griseus), version 1.0. In: In Birds of the World (Schulenberg T. S., Editor). Cornell Lab of Ornithology, Ithaca, NY, USA. [Internet]. 2020. https://doi-org.proxy.lib.ohio-state.edu/10.2173/bow.compot1.01

[96] Marti CD; Poole AF; Bevier LR; Bruce MD; Christie DA; Marks J. Barn Owl (Tyto alba), version 1.0. In: In Birds of the World (Billerman S. M., Editor). Cornell Lab of Ornithology, Ithaca, NY, USA. [Internet]. 2020. https://doi-org.proxy.lib.ohio-state.edu/10.2173/bow.brnowl.01

[97] Fuhlendorf SD, Engle DM. Restoring Heterogeneity on Rangelands: Ecosystem Management Based on Evolutionary Grazing Patterns. Bioscience. 2001;51: 625.

[98] Hovick TJ, Dwayne Elmore R, Fuhlendorf SD. Structural heterogeneity increases diversity of non-breeding grassland birds. Ecosphere. 2014;5. 10.1890/ES14-00062.1

[99] Adams BT, Matthews SN. Diverse temperate forest bird assemblages demonstrate closer correspondence to plant species composition than vegetation structure. Ecography (Cop). 2019;42: 1752–1764. 10.1111/ecog.04487

[100] Rotenberry JT, Wiens JA. Temporal variation in habitat structure and shrubsteppe bird dynamics. Oecologia. 1980;47: 1–9. 10.1007/BF00541768 28309621

[101] Sutherland WJ. Ecological Census Techniques. Cambridge, Uk: Cambridge University Press; 2006.

[102] Sutherland WJ, Newton I, Green R. Bird ecology and conservation: A handbook of techniques. Oxford University Press, Oxford, U.K. 2005.

[103] Bibby C, Burgess N, Hill D, Mustoe S. Bird Census Techniques. 2nd editio. London, UK,: Academic Press Limited; 2000.

