# Supplementary material for: Restoration of gallery forest patches improves recruitment of motacú palms (Attalea princeps) while diversifying and increasing wildlife populations
Source: PLoS One. 2021 Apr 29;16(4):e0250183. doi: 10.1371/journal.pone.0250183 (PMC8084217; doi:10.1371/journal.pone.0250183)
Supplement: S1 Table — (DOCX) [file pone.0250183.s001.docx]

| Variable | PC1 | PC2 |
| --- | --- | --- |
| 1. *Ground Cover PCA* | | |
| bare ground | 0.66 | 0.18 |
| leaf litter | 0.67 | -0.32 |
| grasses | -0.82 | -0.05 |
| forbs | 0.36 | -0.79 |
| deadwood | -0.16 | 0.49 |
| cow dung | 0.73 | 0.55 |
| motacú nuts | -0.63 | 0.52 |
| ferns | 0.09 | -0.82 |
| total plant cover | -0.69 | -0.60 |
| 1. *Shrub Layer PCA* | | |
| motacú | 0.85 | -0.23 |
| other palms | 0.37 | 0.08 |
| broadleaf | 0.46 | -0.72 |
| shrub | 0.49 | 0.74 |
| vine | 0.74 | 0.58 |
| total cover | 0.93 | -0.31 |

**S1 Table.** Factor Loadings for variables on major Principal Component Axes, Principal Component 1 (PC1) and Principal Component 2 (PC2) for (a) the ground cover PCA and (b) the shrub layer PCA.
